# Accurate Real-Time Localization Estimation in Underground Mine Environments Based on a Distance-Weight Map (DWM)

**DOI:** 10.3390/s22041463

**Published:** 2022-02-14

**Authors:** Zhuli Ren, Liguan Wang

**Affiliations:** 1 School of Energy Science and Engineering, Henan Polytechnic University, Jiaozuo 454000, China; 2 Collaborative Innovation Center of Coal Work Safety and Clean High Efficiency Utilization, Jiaozuo 454000, China; 3 School of Resources and Safety Engineering, Central South University, Changsha 410083, China; liguan_wang@csu.edu.cn; 4 Digital Mine Research Center, Central South University, Changsha 410083, China

**Keywords:** underground mine environment, localization, distance-weight map, 3D point cloud, unscented Kalman filter

## Abstract

The precise localization of an underground mine environment is key to achieving unmanned and intelligent underground mining. However, in an underground environment, GPS is unavailable, there are variable and often poor lighting conditions, there is visual aliasing in long tunnels, and the occurrence of airborne dust and water, presenting great difficulty for localization. We demonstrate a high-precision, real-time, without-infrastructure underground localization method based on 3D LIDAR. The underground mine environment map was constructed based on GICP-SLAM, and inverse distance weighting (IDW) was first proposed to implement error correction based on point cloud mapping called a distance-weight map (DWM). The map was used for the localization of the underground mine environment for the first time. The approach combines point cloud frames matching and DWM matching in an unscented Kalman filter fusion process. Finally, the localization method was tested in four underground scenes, where a spatial localization error of 4 cm and 60 ms processing time per frame were obtained. We also analyze the impact of the initial pose and point cloud segmentation with respect to localization accuracy. The results showed that this new algorithm can realize low-drift, real-time localization in an underground mine environment.

## 1. Introduction

Intelligent mining is a development trend and the inevitable choice of mines. All factors point the development of mining enterprises in the direction of intelligent construction, including the safety and efficiency problems of traditional mining, the intelligent upgrading requirements of traditional industries, the market problem of difficult employment in mines, and the rapid development of intelligent technology. One of the main contents of intelligent mining in underground mines is the intelligence of mining equipment. The precise positioning of equipment is the premise of intelligent mining equipment, such as autonomous driving of underground mining equipment, loading and unloading operation, and automatic control of the position of drilling equipment. Therefore, research on high-precision localization technology for underground, confined closed spaces is crucial for the realization of underground intelligent mining.

Ground-based unmanned vehicles, which improve the safety and efficiency of the transportation industry, have been developed rapidly in recent years. Precise localization of the underground mine environment is key to creating greater economic enterprise in the transportation industry. Although GNSS-based localization can achieve centimeter localization accuracy in an open environment, for underground mines, subways, and the like, localization is difficult in underground scenes because of the inability to receive GPS signals [1], variable and often poor lighting conditions, visual aliasing in long tunnels, and airborne dust and water. Low-frequency electromagnetic, ultrasonic sensing measurements, and visual beacon navigation were used for underground localization and continuous tracking in as early as the 1990s [2]. Localization technologies based on Wi-Fi, Bluetooth Low Energy (BLE), Radio Frequency Identification Device (RFID), and Ultra-Wideband (UWB) have been widely used [3,4]. Moridi [5] and Kent [6] used wireless sensor networks and RFID to locate underground mining environments. The position was calculated by the arrival time of the received signal or the difference in the signal strength index. The research of Lavigne and Marshall [7] showed that the localization performance of underground mining scenes usually increases with the increase in beacon density. However, for these localization methods, the corresponding auxiliary localization devices need to be installed in the underground mine environment. With the continuous mining progress, it is necessary to continuously maintain and construct the network. Although the localization accuracy can be improved, many devices and their maintenance are required. At the same time, the rough underground mine environment can introduce cumulative error during the localization process, and more importantly, prevent obtaining a high-precision positioning in the presence of rotation or similar scenes. 

The visual sensor is used for localization and SLAM [8,9,10,11,12] as the camera can provide abundant environmental information, which can be used for the unique identification of localization [13,14,15,16,17,18,19]. However, the study of underground scene localization using vision is relatively scarce. Hurteau [20] used cameras to track autonomous vehicles in underground mines. Kanellakis and Nikolakopoulos [21] proposed a vision-based positioning system for unmanned aerial vehicles (UAV) in a 20-meter roadway environment of underground mines. Jacobson [22] studied the application of ORBSLAM in a large-scale mine environment in 2018. The results showed that the technology could not achieve good localization results in underground environment. At the same time, Jacobson also proposed that monocular cameras could be achieved rough localization with an average error of about 9 m in weak illumination and dusty underground scenes, but it is not applicable to intelligent equipment. Zeng [23] used a camera pointing to the roof of the mine roadway to refine the position, but it needs a continuous and regular vehicle global position estimation system. Kramer [24] proposed that visual inertial sensors could be performed well in the case of almost no ambient lighting, but the test was limited to 170-m-long roadways, and the longitudinal error was between 6 m and 36 m. Dang [25] proposed the thermo-inertial odometry method based on key frames for positioning in a 100 m underground roadway environment, and compared it with the popular laser odometry LOAM [26] proposed by Zhang. The results showed that both methods could achieve a good positioning effect, but this paper did not provide an absolute error evaluation.

There are many studies on laser sensor localization in indoor and outdoor environments [9,11,12,27,28,29]. However, a common limitation discovered within indoor environments is the potential for generating aliased results or large uncertainty in the direction of vehicle motion when traveling within straight corridors, a feature common within underground mines. Madhavan [30] proposed a two-dimensional laser ICP-EKF localization technology for a 150 m underground roadway environment. Chi et al. [31], Nuchter et al. [32], and Dissanayake et al. [33] studied the localization of underground two-dimensional scenes, but did not involve underground three-dimensional scenes. Bakambu and Polotski [34] proposed an autonomous navigation and measurement system based on two inclinometers and gyroscopes for a 150 m underground roadway scene, but the experiment did not provide clear quantitative positioning accuracy results. The large-scale and multi-level mine map construction focuses on high-resolution and surface-scene reconstruction, and the requirements of computational resources are relatively high. Although positioning can be carried out in the process of map construction, this method is only applicable to the positioning of fixed locations, and it is usually not applicable to the real-time position acquisition of underground production equipment such as scrapers. Tabib and Michael [35] proposed a SLAM method for underground mines based on a Gaussian mixture model. The longitudinal error of the system in a relatively small void area (50 m × 40 m) is between 1 and 2 m. Ren [36] put forward the GICP-SLAM method to construct a three-dimensional point cloud map of underground scenes, and carried out relevant experiments in multiple underground scenes. The experiment showed that this method could achieve localization within a certain range, but it is still needed to continue to optimize and improve the location method. Kim and Choi [37] proposed a pattern-matched LIDAR sequential image technique to estimate the location in an underground mine environment. The result showed that the mean absolute error achieved was 0.08 m for the X and Y axes. Another type of positioning method is based on lasers for reactive navigation in underground tunnels. This method used laser real-time detection of roadway intersections and key nodes of equipment walking for navigation. The framework of the entire positioning and navigation process is called “opportunity positioning” [38,39,40,41,42,43,44,45].

The advent of 3D LIDAR has facilitated the precise localization of underground spaces. In unmanned ground projects, LIDAR is also used as the mainstream sensor to achieve high-precision map construction and localization by collecting rich 3D spatial information. Map-based real-time localization is a better choice in underground mining environments. The underground scene map rarely undergoes major changes, relying on the SLAM system for localization while building the map, which wastes computing resources, and it is difficult to ensure the real-time requirements of the system during operation. In this work, we proposed the localization of an underground mine environment both spatially and temporally using a step-by-step approach. Firstly, according to the data acquisition characteristics of 3D LIDAR, a point cloud feature map based on distance weights was constructed. Secondly, the method of point cloud segmentation and initialization positioning is studied, and the influence of roadway scene and initial pose on positioning is explored. Thirdly, the real-time localization of the underground mine environment was realized based on an unscented Kalman filter. In the prediction stage, the position estimation was mainly performed by matching the point cloud frames. In the update stage, the real-time point cloud and the constructed distance-weight map were used to match and modify the predicted position. Finally, experiments were carried out in four underground scenes to demonstrate the feasibility of the method.

The main contributions of this work can be highlighted as follow:

(1) A novel method for the only 3D LIDAR information to obtain accurate 3D localization of the underground mine environment. At the same time, the influence of roadway segmentation and initial position on roadway scene localization was explored.
(2) A novel method for generating an accurate map based on IDW suitable for performing localization in an underground environment.
(3) An evaluation of the proposed approach was tested in four underground scenes, including a smooth straight roadway, smooth loop roadway, rough curved roadway, and rough slope roadway. The localization data can basically cover underground, trackless equipment operation scenes.

The rest of the paper is organized as follows: Section 2 describes the localization method based on DWM, including the algorithm overview, DWM construction, and real-time localization based on the unscented Kalman filter. Section 3 introduces the tests of the underground scenes. Section 4 includes the analyses and discussion of the localization results. Finally, conclusions and recommendations for future work are provided. Our results show that the underground mine environmental localization framework based on the DWM has wider applicability. This work also provides technical support for the practical applications in special underground mine environments such as subways, fire protection, and civil air defense works.

## 2. Algorithm Description

### 2.1. Overview of the Algorithm

The accurate localization framework of the underground mine environment comprised offline DWM construction based on 3D LIDAR, localization initialization, and real-time precise localization based on DWM. 

The construction of the offline map is divided into three parts. Firstly, we construct the point cloud map of the underground scenes based on our previous work, GICP-SLAM [36]. Based on the position, graph optimization theory, the calculated key frame pose, plane constraint, and loop frame were used as constraints in the graph SLAM in the underground mine environment. Secondly, the error characteristics of the point cloud map construction were analyzed. When the laser has a small deflection, the farther the distance of the collected points, the larger the measurement error. At the same time, not all SLAM systems get a completely accurate position. For these reasons, a little jitter will increase the measurement error of the distant data points during the operation of the GICP-SLAM. Finally, we propose a point cloud map correction algorithm based on the inverse distance weighting method. We assigned a weight to each measurement point based on the measurement distance of its 3D LIDAR. In the current frame point cloud, the distance from the far point is less accurate, and that from the closer coordinate point is more accurate. The final output information of this part is the distance weight map, which is used to correct the positioning information during the update stage of the unscented Kalman filter.

For the localization initialization, we constructed a roadway descriptor based on scan context [46] for positioning initialization, which is specially used for the initial pose recognition of underground scenes. The design of the descriptor considered the rotation invariance of environmental information and the processing of noise. At the same time, the key frames in the point cloud mapping are stored with the descriptor and the corresponding KD tree is constructed to facilitate the search. When determining the initial position, the real-time point cloud at the current time is used to find the corresponding final candidate frame and find its corresponding position. Finally, the transformation position between the current frame and the final candidate frame is calculated by point cloud matching method, and the final initial position is determined.

**Figure 1:**
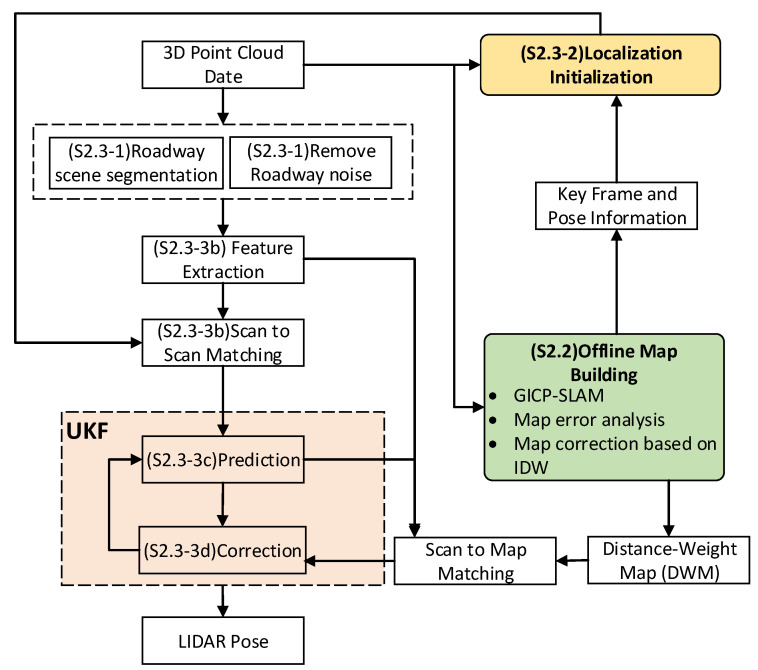
Distance-weight map localization system flow.

For the underground localization based on a distance-weight map, our method is divided into three parts. Firstly, to ensure processing efficiency and localization accuracy, we first carried out noise removal based on roadway segmentation in the point cloud preprocessing stage. Secondly is the point cloud odometer part, which receives the point cloud in the preprocessing stage of the point cloud and calculates the point cloud odometer. The results of the positioning initialization are used as the initial position of the point cloud odometer. Finally, the unscented Kalman filter is used for positioning optimization. This part mainly receives the pose information of the cloud odometer and the map matching pose information. In the prediction stage, we performed the pose estimation by point cloud frame matching. In the update stage, the real-time point cloud and the constructed DWM were used to match and modify the predicted pose, and finally, the real-time precise pose was obtained. At the same time, the pose was also used for the initial pose of the next cycle. The specific process is shown in Figure 1. For ease of viewing, the contents in parentheses in the diagram are the chapter labels for the subchapters.

### 2.2. Offline Map Construction

#### 2.2.1. Point Cloud Map Construction Based on GICP-SLAM

**Figure 2:**
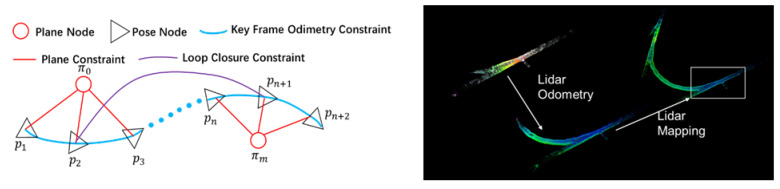
Graph optimization and point cloud map construction diagram. $\left\{p_{1},p_{2},\ldots p_{n+2}\right\}$ is the key frame pose node, and $\left\{\pi_{0},\pi_{1},\ldots \pi_{n}\right\}$ is the extracted roadway ground plane coefficient node.

The point cloud map was constructed based on GICP-SLAM. Based on the position, graph optimization theory, the calculated key frame pose, plane constraint, and loop frame were used as the constraints in the graph SLAM in the underground mine environment. When a frame point cloud is detected as a key frame, the underground environment map needs to be updated. The main method is to convert the real-time point cloud of the key frame into the world coordinate system through coordinate transformation, and compress the point cloud through the octree structure, as shown in Figure 2. For specific details, please refer to the literature we have published [36].

#### 2.2.2. Map Error Analysis

Taking the VLP-16 3D laser as an example, as demonstrated elsewhere [47], the vertical resolution is about 2°, and the horizontal resolution is about 0.4°. A single wire harness as a scan, and a frame cloud of all 16 scans as a sweep. All the points in a frame are scanned serially in sequence. At the same time, only one transmission is sent, followed by one reception. First, from the first angle of the horizontal, generally around 0°, it scans the depth of all 16 points (corresponding to 16 scans) in the vertical direction at this horizontal angle. These 16 points are also serially ordered, and then it goes to the next horizontal angle, such as 0.3°, a horizontal resolution of 0.4°, the next angle of 0.7°, and then 1.1°. When sweeping clockwise, the sweep data are obtained. The output form was XYZ and the intensity information for each point, and no other relationship between points was tracked.

According to the data acquisition characteristics of the 3D laser, when the laser has a small deflection, the farther the distance of the collected points, the larger the measurement error. At the same time, not all SLAM systems get a completely accurate position. For these reasons, a little jitter will increase the measurement error of the distant data points during the operation of the GICP-SLAM. At present, all SLAM systems generally divide the grid into maps during the construction process. According to the continuous increase of real-time point cloud data, the point values in the grid are continuously weighted and averaged, and the mean and variance are obtained. The measurement error is rarely considered. Therefore, in this paper, we assigned a weight to each measurement point based on the measurement distance of its 3D LIDAR. In the current frame point cloud, the distance from the far point is less accurate, and that from the closer coordinate point is more accurate.

**Figure 3:**
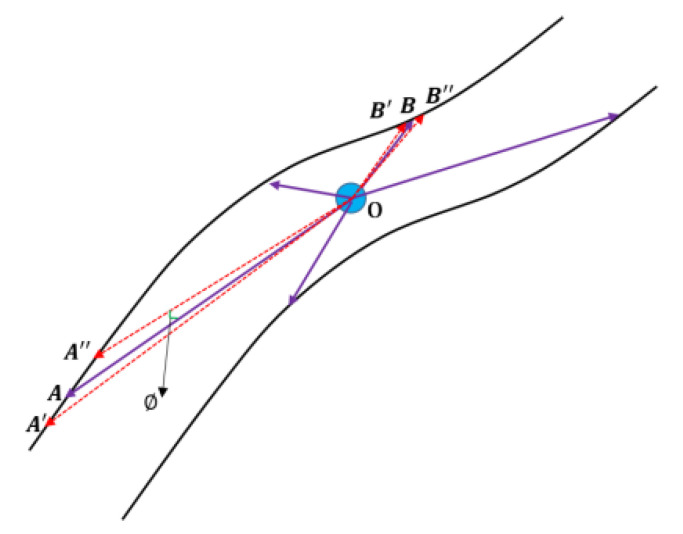
Ranging error description of 3D LIDAR.

Figure 3 is provided to further describe its map error. O is the laser origin. A and B are two measurement points. As can be seen from the figure, the distance of the point A from the origin is farther than the distance of the point B. When the same measurement error angle is ∅, the error of the point A is much larger than the error of the point B. For point A, the error point may be $A^{\prime },A^{\prime \prime }$ in the figure. For point B, the error point may be $B^{\prime },B^{\prime \prime }$ in the figure.

#### 2.2.3. Distance-Weight Map Construction

According to the error analysis of the 3D point cloud map based on GICP-SLAM, we know that the points in the 3D point cloud map in the global coordinate system are obtained by LIDAR position conversion. If the LIDAR position obtained by GICP-SLAM has an error, then the global 3D point cloud map must have an error. Therefore, the distance weight map is a 3D point cloud map corrected by the original 3D point coordinates, and the correction method is to correct the coordinates of the point according to the distance between the point and the current LIDAR center. In the process of point cloud coordinate correction, the point cloud is divided into grids for processing to ensure the processing efficiency and accuracy of the algorithm. The grid size selected in this experiment is 0.1 m. If the grid is too large, the accuracy of the underground scene will be affected, and if the grid is too small, the processing efficiency of the algorithm will be affected.

**Figure 4:**
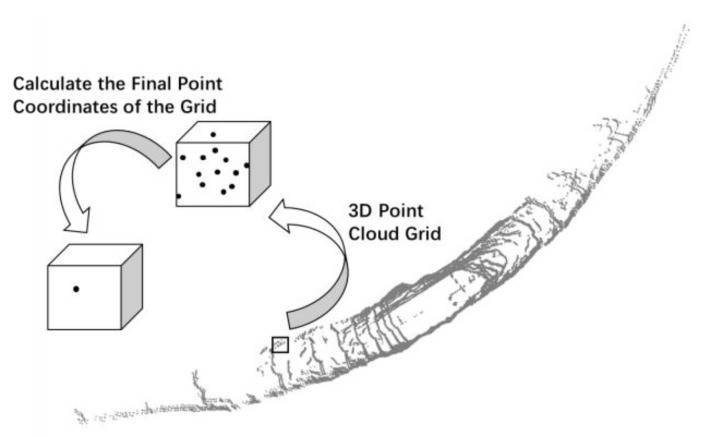
Distance-weight map construction diagram.

**Figure 5:**
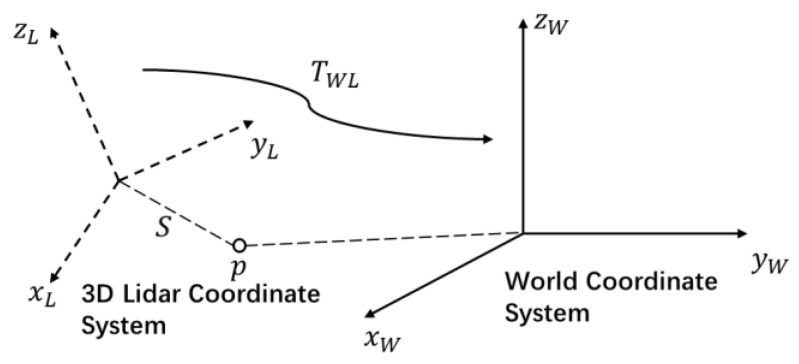
Coordinate system transformation diagram.

In the process of constructing the map, the pose of each coordinate point was corrected to complete the construction of the DWM. As shown in Figure 4, the steps are as follows:

Step 1: According to the obtained 3D LIDAR pose information, the 3D point cloud coordinates in the LIDAR coordinate system were converted to the world coordinate system, as shown in Figure 5. The 3D point coordinate is $p_{L}=(x_{L},y_{L},z_{L})$ in the 3D LIDAR coordinate system, and the 3D LIDAR pose information is represented by the rotation matrix $T_{WL}$; thus, the 3D point cloud coordinates $p_{W}=(x_{W},y_{W},z_{W})$ in the world coordinate system satisfy Equation (1):

(1) $$p_{W}=T_{WL}\cdot p_{L}$$

In the 3D LIDAR coordinate system, we calculated the distance $S=\sqrt{x_{L}^{2}+y_{L}^{2}+z_{L}^{2}}$ from any point in the 3D point cloud to the origin of the 3D LIDAR coordinate.

Step 2: We divided the real-time conversion point cloud and the local map point cloud in the world coordinates into a 3D grid. When the point cloud converted in real time is the first frame data, the point cloud map was initialized. We then divided the first frame transition point cloud into a 3D grid. In the subsequent 3D laser frame, the conversion point cloud and the partial map were divided into a 3D grid. The specific steps are provided as follow: Count the number of point clouds for each grid. According to the measurement characteristics of the laser radar, the measurement error is relatively large at a point far away. According to the calculation method of the distance S from the point to the origin of the LIDAR coordinate system, each point is given a corresponding weight $\omega_{i}=\frac{\frac{1}{S_{i}^{p}}}{\sum_{i=1}^{n}\frac{1}{S_{i}^{p}}}$, and the value of p in this experiment is 2.

According to the converted 3D point coordinates and their corresponding weights, calculate the final point coordinates $p_{WC}=(x_{WC},\;y_{WC},\;z_{WC})$ at this moment in each grid, which satisfies Equation (2):

(2) $$x_{WC}=\sum_{i=1}^{n}\omega_{i}\cdot x_{Wi},y_{WC}=\sum_{i=1}^{n}\omega_{i}\cdot y_{Wi},z_{WC}=\sum_{i=1}^{n}\omega_{i}\cdot z_{Wi}$$

According to the final point coordinates $p_{WC}=(x_{WC},y_{WC},z_{WC})$, calculate its coordinates  $p_{LC}=(x_{LC},y_{LC},z_{LC})$ in the 3D LIDAR coordinate system, which satisfies Equation (3):

(3) $$p_{LC}=T_{WL}^{-1}\cdot p_{WC}$$

Calculate the distance from the origin of the 3D LIDAR coordinate at this moment by Equation (4):

(4) $$S=\sqrt{x_{LC}^{2}+y_{LC}^{2}+z_{LC}^{2}}$$

**Figure 6:**
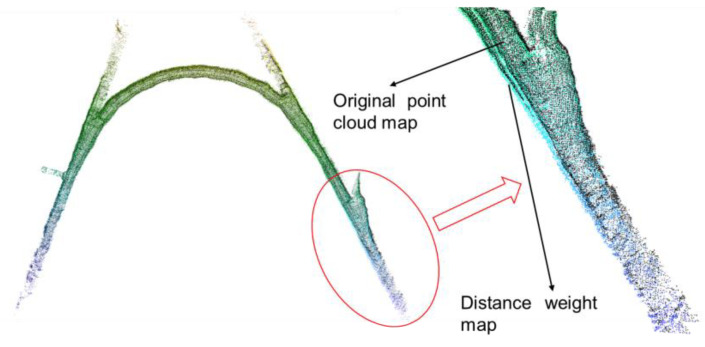
Distance weight map and non-distance weight map comparison.

Step 3: According to the obtained position at each moment and its 3D point cloud information, the first two steps were continuously iterated, and the point cloud map was gradually updated. A complete 3D point cloud map with less alteration from the point cloud noise was obtained, as shown in Figure 6.

### 2.3. Underground Localization

#### 2.3.1. Segmentation of Underground Roadway 

According to the characteristics of the underground roadway, the real-time segmentation of the underground roadway point cloud was carried out. The purpose is to study the influence of roadway roof, roadway floor, and roadway sides on localization, and to remove the influence of point cloud noise.

Assuming that the point cloud obtained in time t is $P_{t}=\{p_{1},p_{2},\ldots ,p_{n}\}$, where $p_{i}$ is the point in point cloud $P_{t}=\{p_{1},p_{2},\ldots ,p_{n}\}$, $P_{t}$ is projected to the range image. Since the horizontal and vertical resolutions of VLP-16 laser radar are $0.2^{{}^{\circ}}$ and $2^{{}^{\circ}}$, the resolution of range image is set to 1800×16. Now, each effective point $p_{i}$ in $P_{t}$ is represented by the only pixel in the range image, and the distance value $r_{i}$ associated with $p_{i}$ represents the Euclidean distance from the point to the 3D LIDAR. 

Point cloud segmentation based on image is that points are grouped into many clusters. Points from the same cluster are assigned a unique label. Point cloud segmentation of the underground roadway environment was divided into roof point cloud, two sides point cloud, and floor point cloud. The application of segmentation to the point clouds can improve the processing efficiency and feature-extraction accuracy. Assuming that the robot runs in a noisy environment, moving objects (e.g., pedestrians, equipment) may form dispersed and unreliable features, because it is unlikely to see the same object in two consecutive scans. To perform fast and reliable feature extraction using a segmented point cloud, the clustering of less than 30 points is ignored. 

After this process, only points that may represent large objects are retained, and only those points are saved in the range image. We also get three attributes for each point: (1) tags for the segmentation points (roof, floor, and two-sides point clouds); (2) column and row indexes in the range image; and (3) the distance value.

#### 2.3.2. Localization Initialization

Since the characteristics of underground scene information are not obvious and similar, the initial problem of localization must be solved first, which will determine whether the subsequent localization can be realized. The similarity between the roof and the floor of the underground roadway is relatively high, and it is difficult to distinguish between the same places. The two sides of the roadway can be distinguished as a feature because of the branch roadway and the pipe cable facilities. Therefore, based on the point cloud segmentation results, the two sides of the roadway are stored in the descriptor, and then the initial pose is accurately determined according to the score calculation between the descriptors and the final candidate frame matching. Based on Scan Context [46], this paper further optimizes the spatial descriptor, which is specially used for the initial pose recognition of underground scenes. The design of the descriptor should consider the rotation invariance of environmental information and the processing of noise. At the same time, the key frames in the point cloud mapping are stored with the descriptor and the corresponding KD tree is constructed to facilitate the search. When determining the initial pose, the real-time point cloud at the current time is used to find the corresponding final candidate frame and find its corresponding pose. Finally, the transformation pose between the current frame and the final candidate frame is calculated by the point cloud matching method, and the final initial pose is determined. Since the real-time localization of the follow-up time is based on the initial pose of the first time, it is necessary to explore the influence of the initial pose on the subsequent localization in the experiment.

#### 2.3.3. Underground Localization Based on an Unscented Kalman Filter

Based on an unscented Kalman filter, the underground localization research was carried out. Firstly, the initial position $x_{0}$ was obtained by the above initial position calculation method. At the same time, the registration results of the two frame point clouds at the adjacent time were used as the position transformation u in the prediction stage, and the matching between the point cloud and the point cloud map at the current time was used as the observation position. Then, based on the unscented Kalman filter system, the pose of the underground equipment was calculated. Specifically, it involves the following four aspects: localization model construction, point cloud registration, prediction model, and update model.

(a) Localization mathematical model construction

**Figure 7:**
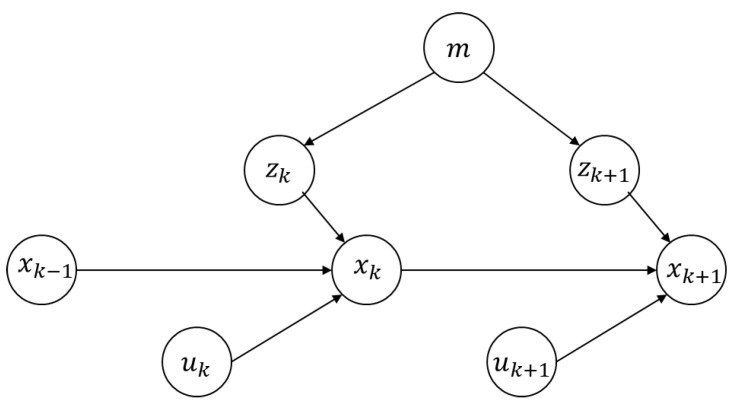
Mathematical representation diagram of the underground localization.

Figure 7 is the positioning diagrammatic drawing of the underground mobile mining equipment. The position of k time is predicted by the $x_{k-1}$ at k−1 time. The prediction process is based on the observation value of k−1 time and k time for the point cloud matching to calculate the transformation at two moments. Then the $x_{k-1}$ is multiplied by the transformation matrix to obtain the predicted position and orientation of k time. The predicted pose is updated and corrected according to the constructed underground point cloud map and the observation value at time k. Finally, the above localization process is repeated to complete the real-time localization.

$x_{k-1}$, $x_{k}$, and $x_{k+1}$ are represented as the posture of underground mining equipment at k−1, k, and k+1 time. $u_{k}$ represents the pose transformation between k−1 and k. $u_{k+1}$ represents the pose transformation between k and k+1. This value can be calculated by the point cloud registration method at two moments. m represents the point cloud map. $z_{k}$ and $z_{k+1}$ represent the observation values at k and k+1 time, which is the return point cloud information of 3D LIDAR.

The above localization process can be expressed by mathematical expressions. There is a motion equation of moving mining equipment from time k−1 to time k, which is expressed by a general mathematical model (Equation (5)):

(5) $$x_{k}=f(x_{k-1},u_{k},w_{k})$$

where $w_{k}$ is the pose noise distribution estimated by the state equation, and function $f(x_{k-1},u_{k})$ is the predicted pose obtained by using the three-dimensional LIDAR odometry.

Corresponding to the motion equation, an observation equation needs to be constructed. This equation describes that mobile mining equipment produces an observation data $z_{k}$ at position $x_{k}$. It is obtained by non-destructive conversion at position $x_{k}$, which is expressed in Equation (6):

(6) $$z_{k}=h(x_{k},v_{k})$$

where $v_{k}$ is the observation noise, and the actual observation value can be obtained by matching the current three-dimensional LIDAR with the point cloud map.

Finally, the localization problem of the underground scene can be summarized as follows: how to solve the position problem of mobile equipment when the position and orientation transformation value $u_{k}$ is known through the matching of the two laser point clouds, and the observation value $z_{k}$ is obtained through the matching of the three-dimensional laser point cloud and the map.

(b) Feature points matching

**Figure 8:**
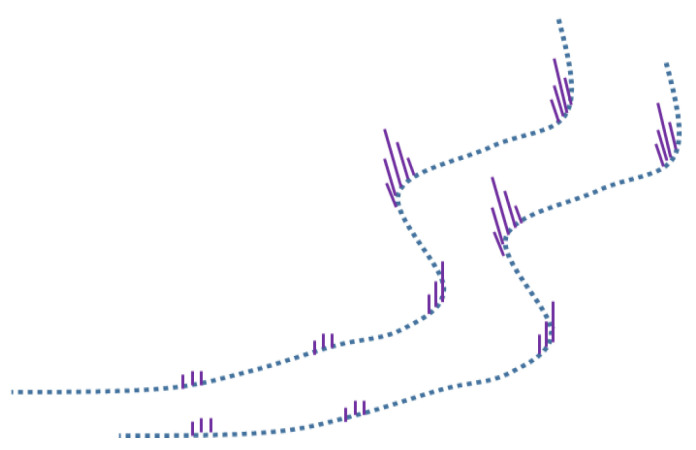
Roadway feature points.

For underground scene localization based on the known points cloud map, the first thing to be solved is the matching problem between the point clouds. With point cloud registration methods (e.g., iterative closest point (ICP), generalized iterative closest point (GICP), and normal distribution transformation (NDT)) it is difficult to obtain accurate positions in underground scenes based on the unscented Kalman filter framework, based on previous studies. Therefore, it is considered to extract the feature points in the underground roadway point cloud for matching and positioning. There are two types of selected feature points: one is edge points and the other is plane points. The selection criteria are determined based on the curvature information of each point, as shown in Figure 8.

Point cloud feature registration is based on the LOAM. When three-dimensional laser is used for underground measurement, the measured points are affected by its measurement mechanism and underlying physical characteristics (such as the size of the laser spot and the measurement noise of the laser), which will lead to the instability of the obtained points. Underground localization needs to select good points, high stability points. Some rules are used to remove the disadvantages of the underground environment, which are described as follow.

In the three-dimensional laser coordinate system, the depth values of each point are calculated. When the depth of the laser point is close to the maximum measurement distance of the three-dimensional laser radar, the error of the measurement results is relatively large. It is necessary to eliminate the point cloud data of this type. The formula of the depth distance of the laser point is in Equation (7):

(7) $$L\left(p\right)=\sqrt{x^{2}+y^{2}+z^{2}}$$

The incident angle of each laser is the angle between the laser beam and the local plane formed by the laser point and the adjacent two laser points. When the incident angle of the laser beam is close to 180° or 0°, the laser point will be stretched very long, resulting in inaccurate measurement results. The calculation formula of the incident angle is in Equation (8):

(8) $$\theta \left(p_{b}\right)=\cos^{-1}\left(\frac{\left(p_{a}-p_{c}\right)\cdot p_{b}}{\left|p_{a}-p_{c}\right|\left|p_{b}\right|}\right)$$

**Figure 9:**
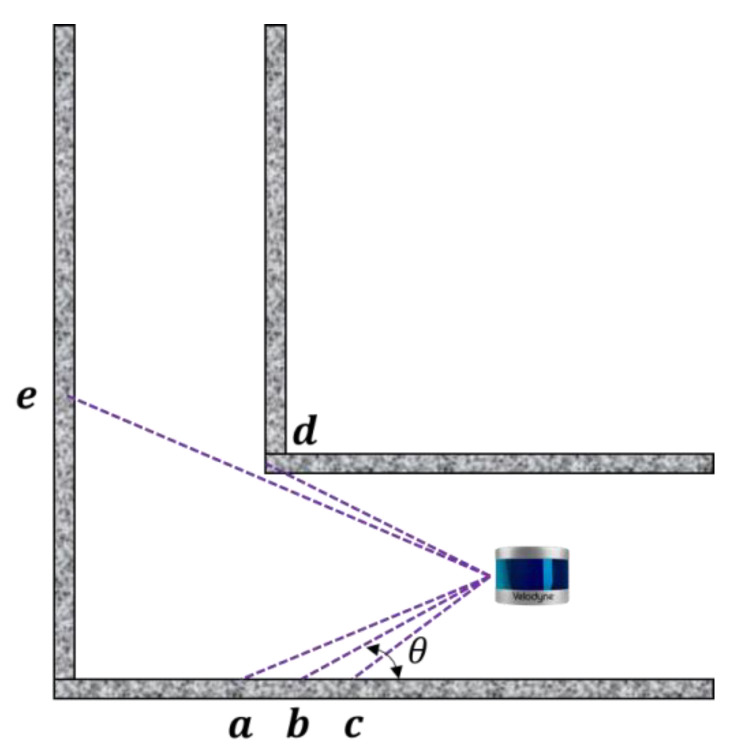
Graphics of the incidence angle reflection angle.

As shown in Figure 9, when two adjacent laser beams are not on the same plane, the plane where one point is located is usually blocked by the plane where the other point is located. The laser points in this case need to be removed. With the continuous movement of 3D laser radar, the points appearing in the previous moment may not exist in the latter moment, which will bring wrong results to laser matching. The specific judgment standard formula is shown by Equation (9):

(9) $$\left|p_{e}-p_{d}\right|\geq 0.1\left|p_{e}\right|,and\;\left|p_{e}\right|>\left|p_{d}\right|$$

(c) Prediction model

Like KF and EKF, UKF predicts and updates the measured values through prediction and state transition matrices, and then derives Kalman gain from the difference between the predicted and measured values. Finally, accurate state vectors and covariances are obtained based on Kalman gain.

The UKF approximates the nonlinear model by defining the sigma point. Because the Jacobian and partial derivatives are not used, the calculation becomes simpler, and the high-order derivative term is not ignored. Therefore, compared with EKF, UKF has less calculation and good effect. The specific flow of the algorithm is that three steps are involved in the prediction: generating sigma points, predicting sigma points state, and predicting mean and covariance. Two steps are used in the update: predicting measurement and updating mean and covariance.

In the prediction model of UKF, the position state vector of the 3D LIDAR is defined by Equation (10):

(10) $$x_{k}={\left[p_{k},q_{k}\right]}^{T}={\left[p_{k}^{x},p_{k}^{y},p_{k}^{z},q_{k}^{0},q_{k}^{1},q_{k}^{2},q_{k}^{3}\right]}^{T}$$

Among them, $p_{k}$ is the three-dimensional coordinates of the 3D laser at time k, and $q_{k}$ is the quaternion of the 3D laser at time k.

In the proposed system, the LIDAR position is first estimated by iteratively applying the extracted feature point matching between successive frames. The real-time input point cloud can be matched with the previous time point cloud frame to obtain the relative pose $\Delta x_{k-1,k}={\left[\Delta p_{k},\Delta q_{k}\right]}^{T}$. At the same time, the position of the LIDAR sensor in the world coordinates at time k−1 is $x_{k-1}$. Therefore, when the point cloud is input at time k, the 3D LIDAR pose at time k can be calculated by Equation (11):

(11) $$x_{k}=x_{k-1}\Delta x_{k-1,k}$$

(d) Correction model

In the correction model of UKF, the 3D LIDAR position is corrected based on the real-time point cloud and the distance-weight map. Firstly, the 3D LIDAR prediction position $x_{k}$ is used as the initial value of the registration, and the pose of the point cloud map registration is $x_{k}^{*}=[p_{k}^{*},q_{k}^{*}{]}^{T}$, which is used as the observation variable $z_{k}^{}=[p_{k}^{*},q_{k}^{*}{]}^{T}$ of the unscented Kalman filter.

## 3. Hardware and Map Scenes

**Figure 10:**
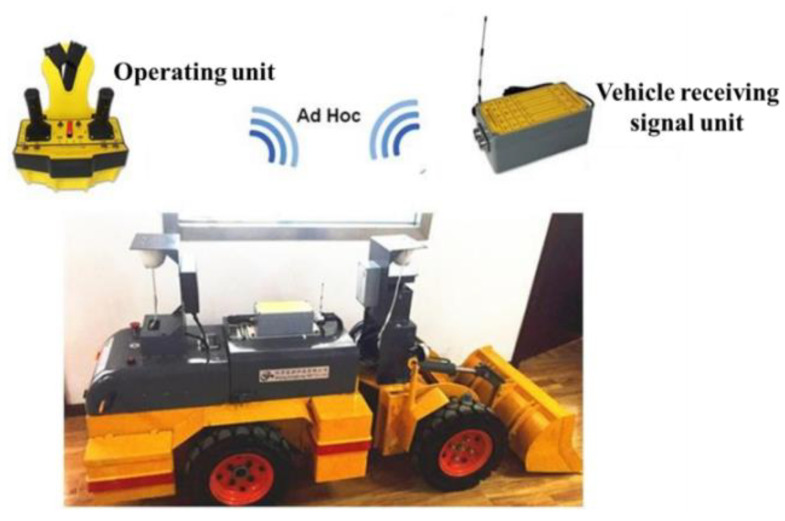
Mobile test platform for underground scenes.

The underground mobile experimental platform used in this experiment consisted of a 16-line 3D LIDAR, mobile power supply, and laptop computer, as shown in Figure 10.

**Figure 11:**
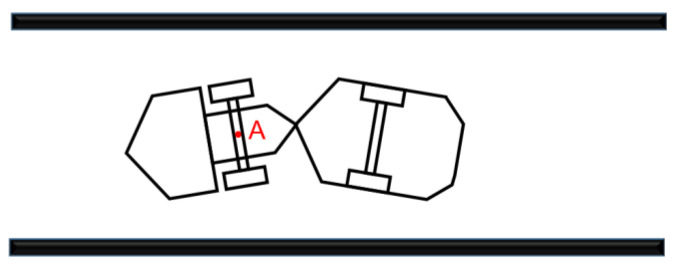
The installation position of 3D LIDAR.

When the mining equipment is working in the roadway environment, due to the hinged structure of the equipment, the driving trajectories of the front and rear bodies are different. In order to realize the positioning of the trackless equipment, a representative point must be selected on the equipment body as the positioning reference point. For most rigid devices, the centroid is usually selected as the reference point for positioning. The mining equipment is a central hinge structure, and the position of its centroid will change with the steering angle and the loading of ore. Therefore, the front center of the mining equipment is selected as the positioning reference point; that is, the intersection of the driving bridge of the front vehicle body and the central axis. As shown in the Figure 11, ***A*** is the center of the driving bridge of the front vehicle body, which is defined as the positioning reference point of the equipment; that is, the installation position of 3D LIDAR.

**Figure 12:**
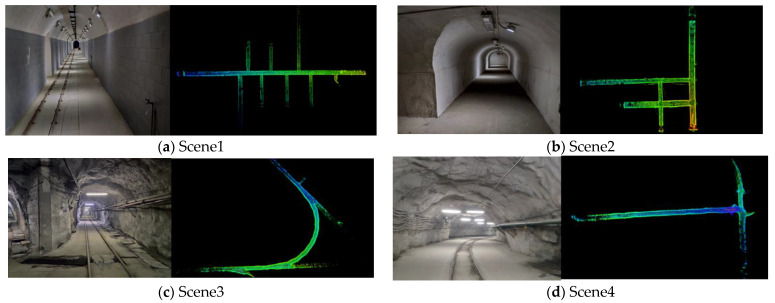
Four underground roadway scene and maps.

We studied four underground scenes: a smooth straight roadway, smooth loop roadway, rough curved roadway, and rough slope roadway, recorded as Scene 1, Scene 2, Scene 3, and Scene 4, respectively (Figure 12). Among them, Scene 1 and Scene 2 were self-built scenes in the laboratory, which were smooth due to the brushed surface. For Scene 3 and Scene 4, the real rough scene of a mine was used, and its feature points were more abundant than that of Scene 1 and Scene 2 and thus theoretically more conducive to localization. The localization algorithm was implemented in C++ and tested on the Ubuntu 16.04 system using an i7-8700 CPU based on a robot operating system.

## 4. Results and Discussion

### 4.1. Trajectory

**Figure 13:**
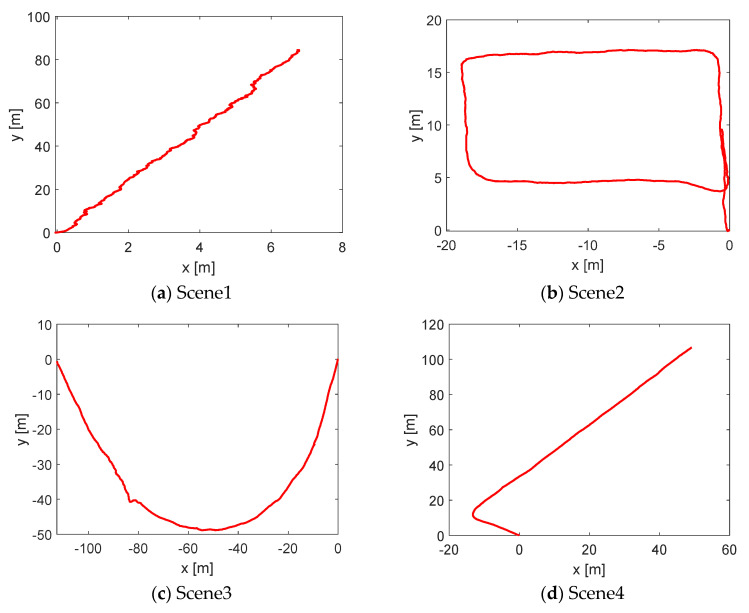
Localization trajectory of the four underground roadway scenes.

According to the four underground roadway scenarios, the real-time localization results and trajectory of the XY plane were obtained, as shown in Figure 13. The localization was analyzed from a qualitative point of view because of walking along the centerline of the roadway during the test. It can be seen that the trajectory curves of the four scenes were consistent with the scene map (in Figure 13).

### 4.2. Error Analysis

**Figure 14:**
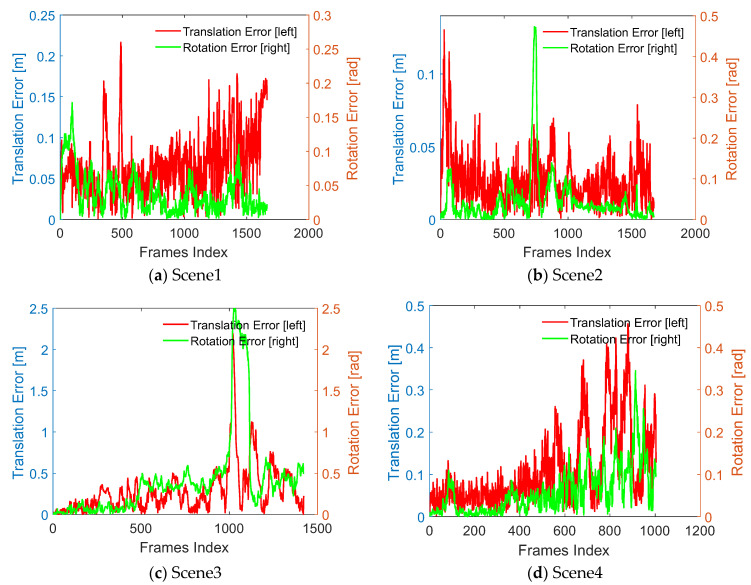
Localization error curve of the four underground roadway scenes.

The actual walking trajectory could not be accurately obtained during the test. When performing the localization error analysis, the trajectory of constructing the DWM was considered as a real trajectory. The translation error (red curve) and rotation error (green curve) with time are shown in Figure 14. The average translation errors of the scenes were 0.0667 m, 0.0266 m, 0.2956 m, and 0.1104 m, and the average rotation errors were 0.0380 rad, 0.0468 rad, 0.5243 rad, and 0.0585 rad, respectively. The overall effect of the four scenarios is better. However, in Scene 3, the localization changed suddenly, resulting in a large deviation. At present, it is considered that the test equipment might have been measured abnormally due to poor contact between the LIDAR and computer. We will continue further testing and analysis to find out the reason for this deviation.

### 4.3. Time Analysis

**Figure 15:**
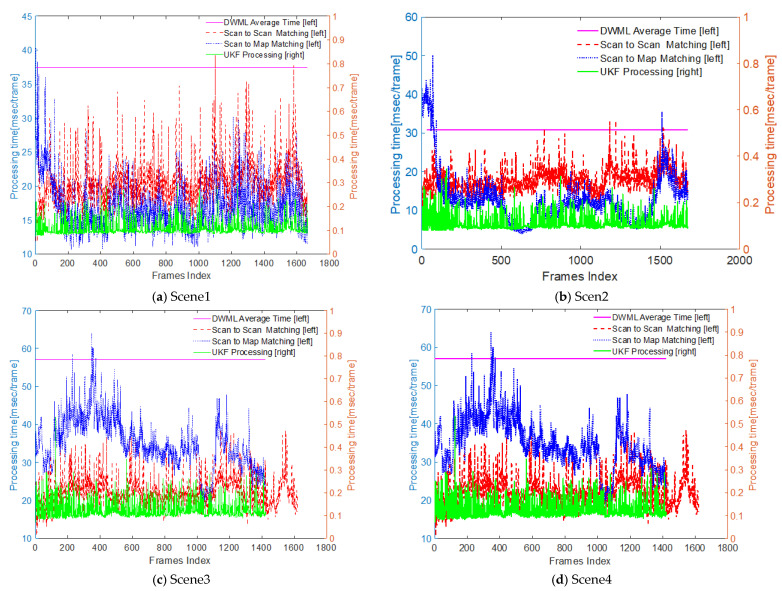
Localization time of the four underground roadway scenes.

To explore the performance of the algorithms based on DWM, we analyzed the running time of the localization algorithm for the four underground scenes, including the matching time in the 3D laser frames, the time of the real-time point cloud and the DWM matching, and the localization correction time based on the unscented Kalman filter. The time curve of the whole localization process is shown in Figure 15. For the maximum, minimum, and average information of each module, Scene 1 and Scene 3 were selected for statistical analysis (Table 1). According to the table, for Scene 1, the matching time between the frame and the frame was more than the matching time of the real-time point cloud and the DWM, while the opposite is true for Scene 3. One of the reasons for that may be the size of the scenes. However, for Scene 1, the total processing time of one frame was 37.4998 ms, while the processing time of one frame was 57.0571 ms in Scene 3. At the same time, because the underground mine environment does not allow a fast driving speed, this system can certainly meet the real-time localization requirement.

### 4.4. Comparative Analysis of Multiple Localization Methods

**Figure 16:**
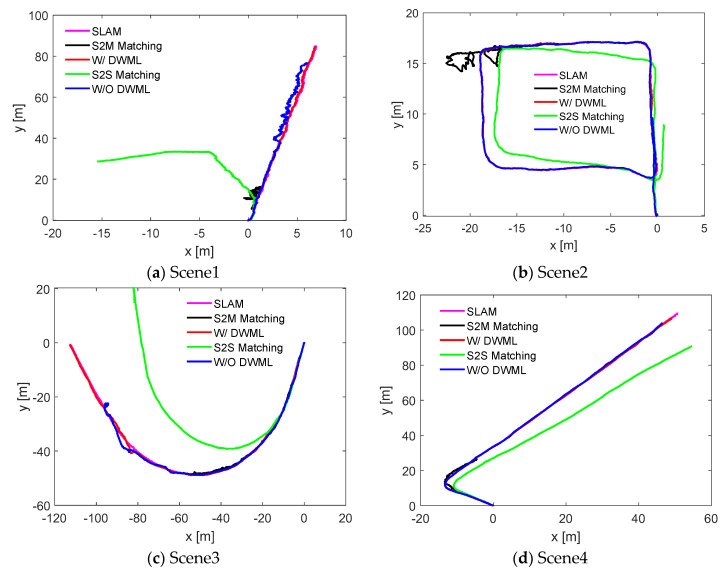
Comparative analysis of the localization methods for four underground roadway scenes.

We further analyzed the localization with DWM, scan-to-scan matching (S2S Matching), and scan-to-map matching (S2M Matching). All the localization results were based on the position of the DWM construction stage (SLAM), as shown in Figure 16. For the four scenarios, the map-based unscented Kalman filter was better than the frame-to-frame matching and frame-to-map matching because, in the underground scene, the presence of few features makes it difficult to obtain accurate results by solely relying on maps or point clouds for registration. At the same time, for the registration between frames, the cumulative error increases with time.

The Root Mean Squared Error (RMSE) was introduced to further analyze the influence of the DWM and the non-DWM on the localization. The smaller the root mean square error, the better the estimation performance of the algorithm. The RMSE is defined by Equation (12):

(12) $$X_{\mathrm{RMSE}}=\sqrt{\frac{1}{n}\sum_{i=1}^{n}{\left(X_{i}-\hat{X}\right)}^{2}}$$

where n is the number of error terms and $X_{i}$ is the i th error term. This error term includes translation error and rotation error. $\hat{X}$ is the average of the error terms; Table 2 lists the root mean square error values for localization with/without the DWM (W DWML or W/O DWML). It can be seen from the table that whether for Scene 1 or Scene 2, the error of using the DWM for localization is smaller than without the DWM localization error; also, the error of localization in Scene 1 is within 4 cm, and the localization accuracy meets the requirements. It further demonstrates the robustness of the localization algorithm based on DWM.

### 4.5. Walking Distance Analysis

**Figure 17:**
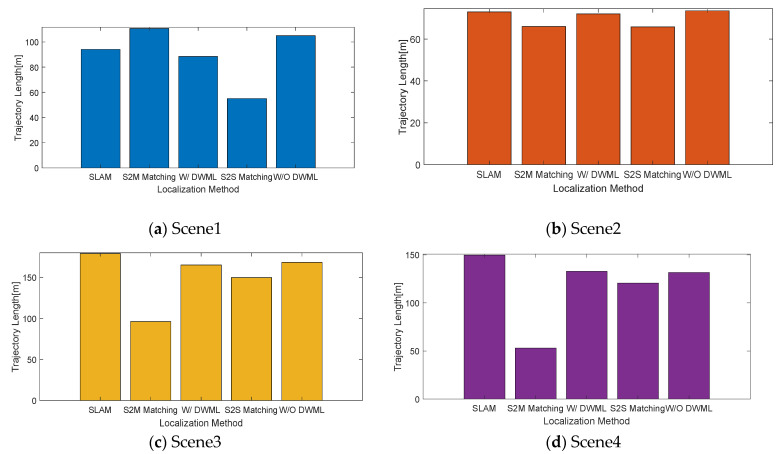
Walking distance of the four underground roadway scenes.

In addition to the analysis of the translation error and rotational error from the localization, the total walking distance in each scene was also considered, as shown in Figure 17. It can be seen from the figure that the effect of localization based on the DWM is the closest to the standard walking trajectory (SLAM), which is superior to the other three localization methods, and the reliability of the localization algorithm is also illustrated.

### 4.6. Analysis of the Initial Position on Localization

**Figure 18:**
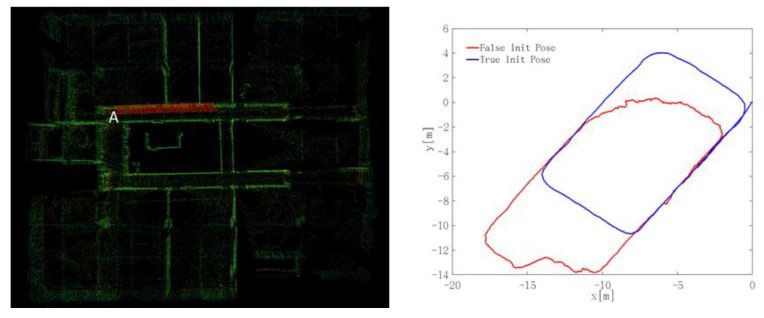
The influence of the initial true coordinate (0, 0, 0) on the localization.

**Figure 19:**
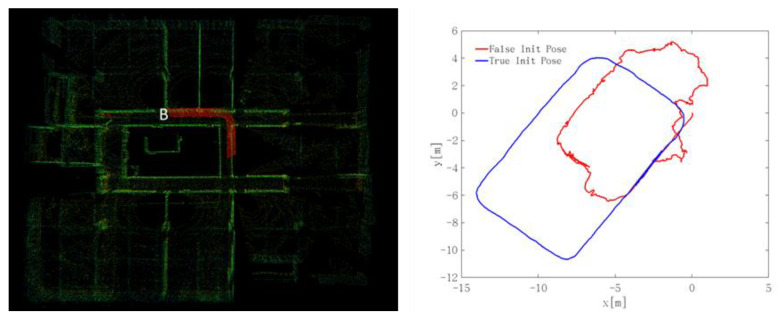
The influence of the initial true coordinate (−3.88, −5.45, −0.17) on the localization.

To verify the influence of the initial position on the localization effect, two starting points A and B were set in the same scene for the experiments. During the test, a real coordinate and an error coordinate were set for each starting point. For the case of Figure 18, the true coordinate of the starting point A was (0, 0, 0) and the error coordinate was (−3.88, −5.45, −0.17). For the case of Figure 19, the true coordinate of the starting point B was (−3.88,− 5.45, −0.17) and the error coordinate is (0, 0, 0).

It can be seen from the trajectory that accurate positioning results can be obtained when the initial point coordinates are true, as shown by the blue curve in the figure. When the coordinates of the initial point are false, it cannot be accurately located, as represented by the red curve in the figure. Therefore, the initial position has a great influence on the real-time positioning, and the wrong initial position will cause the failure of the whole positioning result. Especially for special scenes with less underground characteristics, the determination of the initial position is particularly important. Because of positioning method is based on the initial position, it is continuously calculated forward. If the initial position is not accurate, it will cause the error to accumulate continuously, and the error is difficult to eliminate at one time, which eventually leads to the inaccurate positioning.

### 4.7. Analysis of the Point Cloud Segmentation on Localization

#### 4.7.1. Point Cloud Segmentation Results

**Figure 20:**
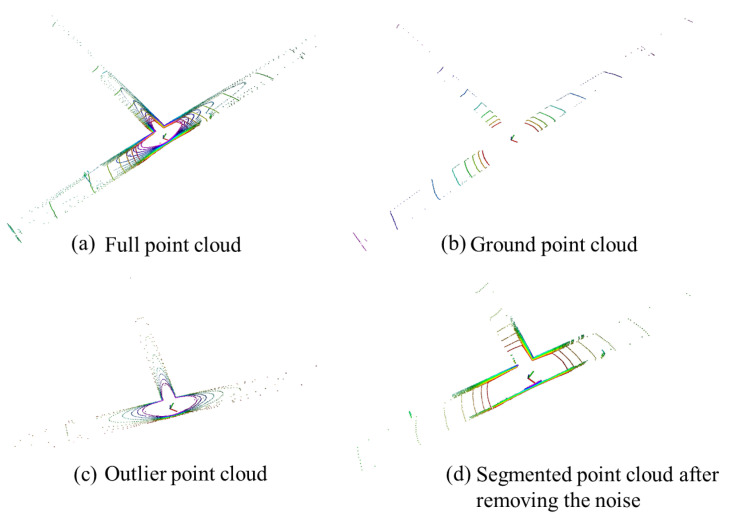
Point cloud segmentation results.

In the underground roadway environment, moving obstacles such as pedestrians will inevitably occur, which will affect the localization results. At the same time, in order to improve the timeliness of the localization while ensuring the localization accuracy, point cloud segmentation is a better method to reduce the number of point clouds and remove the clutter point cloud. As shown in Figure 20, the point cloud is segmented for a frame in underground Scene 2, and then the point cloud of the roadway floor, as shown in Figure 20b, the noise point cloud, as shown in Figure 20c, and the segmented point cloud after removing the noise point cloud, as shown in Figure 20d, are obtained, where Figure 20a is all the point clouds before the point cloud segmentation. The result of the point cloud segmentation is correct for the underground scenes, which is in line with the actual situation.

#### 4.7.2. Comparison of the Localization Results

The positioning experiment was carried out with the segmented point cloud, as well as the final analysis of which part of the roadway point cloud has a greater impact on the positioning results. 

**Figure 21:**
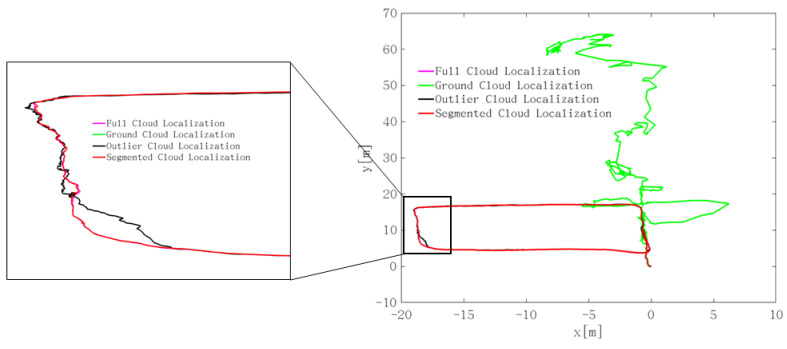
Comparison of the point cloud segmentation on localization.

Figure 21 is the location trajectories of the four kinds of point clouds. It can be seen from the figure that the ground point cloud has a large deviation in localization, and the correct positioning results cannot be obtained. The localization effect of the noise point cloud is better than that of the ground point cloud, but there is a large deviation in the corner. For the whole frame point cloud and noise removal segmented point cloud, a better positioning effect can be obtained. However, under the same localization accuracy, the smaller the number of point clouds used is, the higher the processing efficiency of the algorithm is, which is helpful to improve the real-time performance of the system. Finally, the segmented point cloud with noise removal was selected as the initial point cloud for localization registration.

### 4.8. Discussion

In this paper, we presented a high-precision, real-time, without-infrastructure underground localization method based on 3D LIDAR, which constructs a distance-weight map (DWM) using inverse distance weighting (IDW) and provides a robust estimate of the trajectories of underground mining equipment. The method also includes localization initialization and roadway point cloud segmentation to optimize the positioning results.

The successful implementation of the localization method is of great significance to promote the intelligent process of China’s mining industry. Although this method is only a preliminary exploration of underground intelligent positioning, it is merely the result of the first phase of the intelligent process, and, as such, it is a work in progress. This method has been preliminarily verified in four underground scenes. We further discuss and analyze the positioning results.

The underground mining laboratory built by Central South University and the roadway scene of an underground mine in central China were our testing grounds. For the construction of the distance-weight map, this paper is based on our previous work, GICP-SLAM. Considering the possible influence of positioning error during map construction, a cloud map construction method of underground field attractions based on distance weight was designed. Then the map was used to explore the underground positioning. From the qualitative analysis of the obtained positioning trajectory, the four scenes can achieve the acquisition of pose information. This is a significant improvement within underground mine environments, reducing the average translation error by at least 77%, from 1.32 m to 0.2956 m. We analyzed the real-time problem of positioning. In the underground closed limited scene, mining equipment walking space is very limited, so the real-time positioning is a prerequisite for intelligent equipment mining operations. The positioning time obtained through the experiment is about 60 ms. The current underground manual operation of mining equipment is running at the speed of 10–20 km/h. According to the current positioning time, it can basically meet the requirements. However, there is room to further improve the efficiency of the positioning algorithms. We also compared the positioning effects of the different positioning methods in underground scenes, including the localization with/without DWM, scan-to-scan matching (S2S Matching), scan-to-map matching (S2M Matching), and SLAM. It can be clearly seen from the comparative analysis that the positioning method based on the distance-weight map has better stability for various underground scenes. Therefore, the correction of the map can improve the positioning accuracy, and the distance weight assignment is effective for point cloud map correction.

At the same time, we also found that the initial pose had a great influence on the underground positioning effect. From two experimentation results, when the coordinates of the initial point are false, it cannot be accurately located. It can be seen from Figure 1 that this localization method is based on the initial position in the prediction stage of the unscented Kalman filter. If the initial pose is not accurate, it will cause the error to accumulate continuously, and the error is difficult to eliminate at one time, which eventually leads to the inaccurate positioning. It also proved the feasibility and reliability of the logical relationship of the proposed underground positioning method.

For the special scene of an underground roadway, we segmented the roadway scene. The purpose was to study the influence of the roadway roof, roadway floor, and roadway sides on localization, and to remove the influence of point cloud noise and optimize the calculation efficiency of underground localization. We found that a better positioning effect can be obtained for the whole frame point cloud and noise removal segmented point cloud. However, under the same localization accuracy, the smaller the number of point clouds used is, the higher the processing efficiency of the algorithm is, which is helpful to improve the real-time performance of the system. Finally, the segmented point cloud with noise removal was selected as the initial point cloud for localization registration.

## 5. Conclusions and Future Work

In this study, we proposed a high-precision localization system based on 3D LIDAR to operate with the complex conditions found within the underground mine environment. The distance-weight map was developed based on inverse distance weighting (IDW) to implement error correction. The proposed method fused the only LIDAR and DWM date within an unscented Kalman filter to achieve a reliable localization estimate.

We evaluated our proposed localization method with data captured from four underground scenes. We demonstrated an average translation error of 0.0667 m, 0.0266 m, 0.2956 m, 0.1104 m and rotation error of 0.0380 rad, 0.0468 rad, 0.5243 rad, and 0.0585 rad for the four underground scenes. The localization root mean squared error of 4 cm and the localization time of about 60 ms were obtained. This is a large improvement for underground mine environments.

It is important to note that the initial pose has a lager influence on the real-time positioning, and the bad initial pose may cause the failure of the whole positioning result. We also presented a computational analysis of the point cloud segmentation on localization. The segmented point cloud with noise removal is more favorable to the localization accuracy and processing efficiency of the proposed algorithm.

To further improve the localization accuracy of the underground scene, the factors affecting the localization of the DWM need to be considered for the actual production in which subsequent applications can be better applied. At the same time, sensors such as IMU need to be considered for addition to the system for multi-sensor Fusion localization, which reduces the cumulative error caused by the localization process. Since the initial pose was set as the coordinate origin, it was further improved. Rapid identification and localization need to be further studied.

## Figures and Tables

**Table 1:** Process time of each module in Scene 1 and Scene 3.

	Scene1			Scene3		
	Mean/ms	Max/ms	Min/ms	Mean/ms	Max/ms	Min/ms
**Scan to Scan Matching**	20.4992	39.2756	11.7567	22.1333	38.4930	10.7109
**Scan to Map Matching**	16.8920	40.3859	10.6786	34.7994	64.0313	17.8489
**UKF Processing**	0.1086	0.2973	0.0664	0.1245	0.5322	0.0693
**DWML Average Time**	37.4998			57.0571		

**Table 2:** Root mean square error value of Scene 1 and Scene 3.

Localization Algorithm	Scene1 RMSE		Scene3 RMSE	
	Trans./m	Rot./rad	Trans./m	Rot./rad
**W/DWML**	0.0362	0.0292	0.2956	0.5243
**W/O DWML**	0.2467	1.3562	0.6676	0.2641

## Data Availability

Not applicable.
